# Prevention of Edema After Coronary Artery Bypass Graft Surgery by Compression Stockings

**DOI:** 10.5812/cardiovascmed.17463

**Published:** 2014-04-01

**Authors:** Alireza Alizadeh-Ghavidel, Parisa Ramezannejad, Yalda Mirmesdagh, Ali Sadeghpour-Tabaei

**Affiliations:** 1 Heart Valve Disease Research Center, Rajaie Cardiovascular Medical and Research Center, Iran University of Medical Sciences, Tehran, IR Iran; 2 Rajaie Cardiovascular Medical and Research Center, Iran University of Medical Sciences, Tehran, IR Iran

**Keywords:** Stockings, Compression, Coronary Artery Bypass, Edema

## Abstract

**Background:**

Lower limb edema may occur after removal of the saphenous veins in coronary artery bypass graft (CABG) surgery. Compression therapy is often used to prevent postoperative edema.

**Objectives:**

The objective of this study was to evaluate the efficacy of medical compression stockings (TED) on the prevention of donor limbs edema and wound complications after CABG surgery.

**Patients and Methods:**

In this prospective cohort study, we enrolled 100 patients who underwent elective CABG surgery at Rajaie Cardiovascular Medical and Research Center. The patients were divided into two groups; group A who applied TED stockings regularly (exposure group) and group B who did not apply TED stockings at all or apply it irregularly (no exposure group). The degree of donor limb edema and the differences of the peripheries of calf and thigh before and after the surgery (in 1, 2 and 4 weeks) were recorded and analyzed statistically.

**Results:**

The patients' weight (P = 0.02) and the degree of their daily activity (P = 0.002) were the significant factors for the incidence of the donor limbs edema. The incidence and degree of lower limb edema were significantly lower in exposure group 4 weeks after the surgery (P < 0.001). The differences of the periphery of the calf before (at admission time) and after the surgery (in 1, 2 and 4 weeks) between two groups were also statistically significant (P = 0.41, P = 0.39, P = 0.40, respectively). Lower limb wound complications was higher in patients who have peripheral edema in the 4th week of post-CABG (P = 0.09).

**Conclusions:**

Regular use of TED stockings may have positive effects on the prevention of donor limb edema (especially higher degrees of edema) and wound complications after CABG surgery.

## 1. Background

Coronary artery disease is a global epidemic and the most common cause of death among non-infectious diseases (1). The number of coronary artery bypass graft (CABG) surgeries has been increased recently and it is estimated to be 800,000 surgeries worldwide annually (2). This procedure is performed by harvesting saphenous vein or radial artery. Besides arterial grafts, removal of the saphenous leg veins is the most common procedure in CABG (3-5).

There are different complications at the operation site, including, edema, erythema, tenderness and infection (6-9). Edema is a well-known complication in patients following CABG surgery and may cause discomforts in the donor legs, such as numbness, weakness, immobilization, sleep disorder and delayed healing (10-12).

 Compression therapy is the most common intervention to treat venous and lymphatic vessels disorders (13). This technique suppresses postoperative pain and edema by preventing the formation of hematomas along the course of the removed vein. Compression improves venous return of the lower extremities and therefore speeds-up the healing process (14). Based on the previous studies, the medical elastic compression stocking is one of the most frequent used medical compression techniques during prophylactic and maintenance phase of lower limb edema due to its ease of use and therapeutic function (15). As a result, one of the most important approaches for suppressing lower limb edema after CABG surgery is compression therapy with medical elastic compression stockings. However, few studies have been conducted to evaluate the beneficial effects of elastic stockings on the prevention and treatment of lower limb edema in Iran (16).

## 2. Objectives

This research study aimed to evaluate the effect of medical elastic compression stockings on the prevention of the lower limb edema of patients underwent CABG surgeries.

## 3. Patients and Methods

After the approval of the study, the protocol was granted by the institutional review board. One-hundred patients (79 men and 21 women, with mean age of 60 ± 9 years), were enrolled in this prospective cohort study. They were scheduled for elective CABG surgery at Rajaie Cardiovascular Medical and Research Center, Tehran, Iran from February 2011 to January 2012, and written informed consent was obtained from each participant. Demographic data, including age, gender, height, weight, medical history (e.g. diabetes mellitus) and drug history (e.g. diuretics) of the patients were collected using questionnaire method.

 In the next step, by preoperative methods like physical examination or duplex ultrasound report, patients with deep vein thrombosis (DVT), varicose veins and lymphadenopathy were excluded. Also, the patients who suffered from extreme generalized edema (anasarca) before or after surgery (due to different reasons such as heart failure, transient nephropathies and DVTs) and the cases on high dose of diuretics (because of significant heart failure) were excluded from the study.

Techniques used to remove saphenous vein were called the open-surgical-incision direct approach. The anatomical locations of the vessels taken for graft depend on the vein size and patient's characteristics. In routine clinical practice, elastic bandages were applied over the sterile dressing on the donor limbs immediately after the surgery. Twenty-four hours later, the initial dressings were removed in the ICU, and the wounds were cleaned and sterile dressings were applied again. Next, a pressure dressing and thrombo embolic deterrent (TED) -above knee stockings- were applied to the donor limbs. The patients were asked to remove TED stockings every night and wore it the next morning.

The patients were evaluated regarding the method of saphenous vein harvesting, postoperative cardiac events and the incidence of complications in the donor leg. At the end of the fourth week post-operation, the patients were also evaluated regarding their daily activity (routine daily chores, irregular and regular exercising), use of low sodium diet (diets restricted to foods with naturally low sodium content and prepared without added salt) and TED stockings.

The cases were divided into two groups based on the way they applied TED stockings. Fifty patients who applied TED stockings routinely and regularly were put in group A (exposure group) and the 50 patients who did not apply TED stockings regularly or never use it, were put in group B (no exposure group).

The existence of donor leg edema after CABG surgery was considered the subject of this study. The degree of the donor leg edema is scored as follows: 0; no edema, +1; barely detectable impression when finger is pressed into skin, +2; slight indentation, 15 seconds to rebound, +3; deeper indentation, 30 seconds to rebound, +4; > 30 seconds to rebound (17). The periphery of the calf (the largest circumference of the calf), and the periphery of the thigh (the periphery of the thigh 10 cm above patella) were measured by tape and recorded in 4 stages; before the operation (at surgery ward), 1 week after operation (at surgery ward, before discharge), 2 weeks and then 4 weeks later (at Rajaie heart clinic, during follow up period) in lying position for all the patients.

 The differences of the peripheries of calf and thigh before the surgery and 1, 2 and 4 weeks after the surgery were measured and recorded for all cases. The acquired data were analyzed by SPSS 15 for Windows (SPSS Inc., Chicago, Illinois) and the statistical tests were carried out including: Student's t-test Mann Whitney U test, and chi-squared test. P value ≤ 0.05 was considered statistically significant.

## 4. Results

Lower limb edema was occurred 4 weeks after the surgery in 86 cases (with mean age of 61 ± 9 years, height: 168 ± 8 cm, weight: 75 ± 11 kg) out of 100 patients. There were 66 (77.3%) male cases and 20 (23.2%) female patients in this group. Among 16 patients (with mean age of: 59 ± 10 years, height: 164 ± 8 cm, weight: 67 ± 12 kg) with no lower limb edema, 10 were male and 6 patients were female. There was no significant difference regarding the gender of the patients between two groups (P = 0.67). The mean age, height and weight of the patients were compared between these two groups (edema, no edema) and just the mean weight of the patients were significantly higher in group edema (P = 0.47, P = 0.20, P = 0.09, respectively). The patients were also compared regarding the degree of their daily activity; 60 patients out of 84 patients with edema (71.4%) performed daily chores versus 8 patients out of 16 patients without edema (50%) also 21 cases (25%) in edematous patients exercised irregularly versus three (18.8%) in the patients without edema, and finally 5 patients (31.3%) exercised regularly in the patients without edema versus three cases (3.6%) in the other group. This variable was significantly different between two groups (P = 0.002). Fourteen patients (16.7%) with lower limb edema 4 weeks after the surgery and one patient (6.3%) without edema 4 weeks after the operation had a history of diuretic intake (P = 0.07). Thirty five patients (41.7%) who suffered from edema 4 weeks post-op and 8 cases (50%) with no edema at this time had a history of diabetes mellitus (P = 0.93). All the patients with no edema had considered low sodium diet during the time of the study in comparison with 81 cases (96.4%) with edema who also considered low sodium diet (P = 0.06). Postoperative cardiac events (MI, heart failure, and decrease in EF more than 10%) did not occurred in patients without edema. However, two patients (2.4%) with edema suffered from postoperative cardiac complications (P = 0.57). Wound complications (ecchymosis, infection) at the site of saphenous vein harvesting occurred in 12 patients (14.3%) with edema while there was no case of wound complication in patients without peripheral edema 4 weeks after the surgery (P = 0.09). The method of saphenous vein harvesting was also compared between the two groups 4 weeks post-op and the difference was not statistically significant (Table 1). The patients were also divided based on the way they applied TED stockings: 50 patients were in group A (the exposure group who applied TED stockings regularly) and 50 patients were in group B (no exposure group who applied TED stockings irregularly). Baseline characteristics were compared between two groups and the results have been demonstrated in Table 2. The distribution of donor limbs and the incidence of lower limb edema 1, 2 and 4 weeks after the surgery were compared between 2 groups and the results have been shown in Tables 3 and 4. The degree of the edema was statistically different between two groups in 1, 2 and 4 weeks after the surgery (Table 5). The difference of the periphery of the calf before (at admission time) and after the surgery (in 1, 2 and 4 weeks) between two groups was statistically significant (P = 0.004, P < 0.001, and P < 0.001 respectively). However, the difference of the periphery of the thigh before (at admission time) and after the surgery (in 1, 2 and 4 weeks) between two groups was not statistically significant (P = 0.41, P = 0.39, P = 0.40, respectively).

**Table 1.** The Comparison of Distribution of Donor Limbs (Saphenous vein Harvesting) in Patients With Edema and no Edema 4 Weeks After the Surgery ^a^

Donor Limb (s)	Patients With Edema (n=84)	Patients Without Edema (n=16)
**Right calf**	3 (3.6)	0
**Left calf**	20 (23.8)	7 (43.8)
**Right calf + right thigh**	18 (21.4)	2 (12.4)
**Left calf + left thigh**	37 (44.0)	7 (43.8)
**Both calves**	3 (3.6)	0
**Both calves + one thigh**	3 (3.6)	0

^a^ Data are presented as count (%), P value is 0.39.

**Table 2.** The Comparison of Baseline Characteristics Between Two Groups of Patients ^a^

	Compression Stocking Regularly Group (n=50)	Compression Stocking Irregularly Group (n=50)	P value
**Gender**			0.57
Male	36	43	
Female	14	7	
**Age, y**	59 ± 9	61 ± 9	0.25
**Weight, kg**	72 ± 13	75.2 ± 10	0.20
**Height, cm**	166 ± 8	169 ± 8	0.12
**Diabetes mellitus **	20 (40)	23 (46)	0.33
**Degree of daily activity **			0.03
Daily chores	28 (56)	40 (80)	
Irregular exercising	16 (32)	8 (16)	
Regular exercising	6 (12)	2 (4)	
**Diuretic use**	8 (16)	7 (14)	0.91
**Low-sodium diet use**	49 (98)	48 (96)	0.60

^a^ Data are presented as count (%) or mean ± SD.

**Table 3.** The Comparison of Distribution of Donor Limbs in Two Groups of Patients ^a^

Donor Limb (s)	Compression Stocking Regularly Group (n=50)	Compression Stocking Irregularly Group (n=50)
**Right calf**	1 (2)	2 (4)
**Left calf**	13 (26)	14 (28)
**Right calf + right thigh**	10 (20)	10 (20)
**Left calf + left thigh**	23 (46)	21 (42)
**Both calves**	2 (4)	1 (2)
**Both calves + one thigh**	1 (2)	2 (4)

^a^ Data are presented as count (%), P value is 0.718.

**Table 4.** The Incidence of Lower Limb Edema (calf and tigh) in 1, 2 and 4 Weeks After the Surgery in Two Groups of Patients ^a^

	Compression Stocking Regularly Group (n=50)	Compression Stocking Irregularly Group (n=50)	P value
**One week after surgery**			0.23
Edema	47 (94)	49 (98)	
No edema	3 (6)	1 (2)	
**Two weeks after surgery**			0.08
Edema	46 (92)	49 (98)	
No edema	4 (8)	1 (2)	
**Four weeks after surgery**			0.04
Edema	35 (70)	49 (98)	
No edema	15 (30)	1 (2)	

^a^ Data are presented as count (%).

**Table 5.** The Comparison of Different Degrees of Edema in 1, 2 and 4 Weeks After the Surgery in Two Groups of Patients ^a^

Degrees of Edema	Compression Stocking Regularly Group (n=50)	Compression Stocking Irregularly Group (n=50)	P value
**One week after the surgery**			0.02
0	3 (6)	1 (2)	
+1	18 (36)	5 (10)	
+2	17 (34)	32 (64)	
+3	12 (24)	12 (24)	
+4	0	0	
**Two weeks after the surgery**			0.07
0	4 (8)	1 (2)	
+1	23 (46)	7 (14)	
+2	15 (30)	35 (70)	
+3	8 (16)	7 (14)	
+4	0	0	
**Four weeks after the surgery**			< 0.001
0	15 (30)	1 (2)	
+1	22 (44)	20 (40)	
+2	13 (26)	26 (52)	
+3	0	3 (6)	
+4	0	0	

^a^ Data are presented as count (%).

## 5. Discussion

Coronary artery disease is a global epidemic and the number of CABG surgeries has been increased recently. One of the well-known complications in patients following CABG is edema in the donor leg. Compression therapy is the most frequent intervention to reduce postoperative edema.

 In this study we evaluated the effects of TED compression stockings on the prevention of postoperative lower limb edema. The demographic data including age, gender and height of the patients had no relation to the incidence of edema 4 weeks after the surgery. However, there was a significant relation between weight and the incidence of edema in patients. This result proved that edema occurred more in obese patients and in contrast to the study of Khoshgoftar et al. age was significantly associated with the incidence of edema (16). 

In our study the cases with more daily activity suffered significantly less from lower limb edema. However, Khoshgoftar et al. concluded in their study that changing leg posture, being out of bed and walking 24 hours after the operation and lower limb physiotherapy had no significant effect on donor limb edema (16). Diuretic use and the history of diabetes mellitus had no effect on the incidence of lower limb edema 4 weeks after the operation, similar to the studies of Khoshgoftar et al. (16) and Belczak et al. (18). Our study also revealed that there was no relation between the low sodium diet and the incidence of edema 4 weeks post-op.

 The incidence of wound complications at the site of saphenous vein harvesting had no effect on the incidence of the lower limb edema in our study. In the study of Belczak et al. the incidence of suture infection at lower limb after CABG surgery was reported 25%; however, its relation with the incidence of edema was not evaluated (18). There was no relation between the method of saphenous vein harvesting and the incidence of edema four weeks after the surgery. In the similar study, the effect of the number and the length of the surgical incisions on the incidence of lower limb edema were evaluated and concluded that these factors had no effect on the incidence of postoperative edema (18).

In this study, the incidence of edema 1, 2 and 4 weeks after the surgery was compared between two groups of patients (with TED stockings, without TED stockings). The incidence of edema 1 and 2 weeks after the surgery was not significantly different between two groups. However, lower limb edema was occurred most significantly in patients with no TED stockings four weeks after the operation. The results revealed that regular applying of TED stockings after the operation had no effect on the prevention of lower limb edema 1 and 2 weeks after the operation, but may have a significant effect on the prevention of edema formation 4 weeks after the surgery. In the similar study, the incidence of edema was not evaluated at different time intervals. However, the study conducted by Khoshgoftar et al. showed the effectiveness of TED stockings in edema prevention of the foot and the heel regions (16).

In this study, the difference of the periphery of the calf before (at the admission time) and after the surgery (1, 2 and 4 weeks) between the two groups was statistically significant. Therefore, TED stockings had a significant effect on the prevention of edema formation in the calf region but no significant effect on the thigh region. In the similar study by Khoshgoftar et al. the differences of the periphery in the foot and the heel area at admission time and discharge time (12 days after the operation) were significant (16). In the present study wound complications in saphenous vein harvesting site was significantly lower in patients without peripheral edema 4 weeks after CABG surgery; hence, TED stockings may have a positive impact on this outcome.

Based on the results of this study, it seems that TED stockings have significant effect on prevention of edema formation in donor leg, four weeks after the surgery (CABG). It may also have significant influence on decreasing the rate of wound complications and also the degree of donor limb edema 1, 2 and 4 weeks after the operation.

 The limited number of patients and lack of randomization can affect the results. Besides, we had to rely on the patients regarding the regular use of TED, low salt diet and the degree of physical activity.
